# Statement of Retraction: 18β-Glycyrrhetinic acid inhibits the apoptosis of cells infected with rotavirus SA11 via the Fas/FasL pathway

**DOI:** 10.1080/13880209.2022.2142339

**Published:** 2022-11-08

We, the Editors and Publisher of *Pharmaceutical Biology*, have retracted the following article:

Authors: Xiaoyan Wang, Fang Xie, Xiaofeng Zhou, Ting Chen, Ye Xue, and Wei Wang

Article: 18β-Glycyrrhetinic acid inhibits the apoptosis of cells infected with rotavirus SA11 via the Fas/FasL pathway

Journal: Pharmaceutical Biology

Volume 59; 2021; Pages 1096–1103; DOI: 10.1080/13880209.2021.1961821

The authors noticed that some of the graphs and controls in Figure 3A and Figure 4A were similar. Upon further investigation, the authors realised they had made errors when creating the graphs and figures, leading to inconsistent results. In view of these problems, the authors contacted the journal to request a retraction.

We have been informed in our decision-making by our policy on publishing ethics and integrity and the COPE guidelines on retractions.

The retracted article will remain online to maintain the scholarly record, but it will be digitally watermarked on each page as “Retracted”.

